# Good medical practice in a time of chronic disease: time to retrace our steps?

**DOI:** 10.7189/jogh.06.020301

**Published:** 2016-12

**Authors:** Anand Bhopal

**Affiliations:** University College London, London, UK

Across the world, a wide range of non-communicable diseases, drug resistant infections and infectious ailments flourish. Yet the ability of doctors to tackle the multiple morbidities of patients is increasingly limited. I believe this is part due to the ill–defined role of doctors in tackling population health issues such as the structural determinants of health which shape the social conditions in which disease thrives. Here I draw on historical examples to put forward the case that medicine needs to re–envisage the role of the doctor and put greater value on preventative measures. I use the example of the National Health Service (NHS) in the United Kingdom to discuss the challenge and opportunities of uniting clinical practice and public health in the common goal of addressing today's greatest health challenges.

In the midst of great technological advances and societal change worldwide, the role of doctors, and health care more broadly, within broader health systems is increasingly unclear. In high– and low–income settings alike traditional medical approaches are of limited effectiveness in reversing the rise in non–communicable diseases, however, much of the discourse continues to be grounded in the doctor–patient relationship, with little mention of broader population health needs. Doctors through their position as health care providers and role as advocates have great opportunities to address the social determinants of health, however, the precise role and responsibility of doing so remains ill–defined. The nature of modern health care is such that we simultaneously increase prescriptions of costly medications and broaden access to high–tech interventions, yet do little to tackle the social milieu in which disease flourishes. Over recent years an array of health problems ranging from burgeoning childhood obesity rates to emerging infectious diseases and antimicrobial resistance have developed which a variety of long term consequences with poor prospects for curbing downstream effects.

Reversing such trajectories needs action at all levels, aligning efforts across primary care, secondary care and public health, both within and between nations. At a time when disease is increasingly driven by macro–level determinants (eg, non–communicable diseases) and collective action problems (eg, antimicrobial resistance), there is a growing need for doctors to take greater consideration of, and responsibility for, population health. This is not just a responsibility of public health professionals. Tackling issues such as antimicrobial resistance relies addressing the challenges faced on the individual clinician level as well as the overarching systems which perpetuate the problem. It is clear from the responses of the Royal Colleges to Strategic review of health inequalities in England post–2010 (*Fair Society, Healthy Lives*) that there is overwhelming agreement on the institutional level that action should be taken by doctors, yet little focus on how [1]. Social determinants of health and the inequalities which result must be seen as fundamental to what doctors do, not an insulted occurrence distinct from medicine. Fundamentally this ambition hinges on ensuring medical training is designed to meet today’s population health needs. I believe it is time to re–trace our steps and learn from the past in order to address the challenges ahead.

**Figure:**
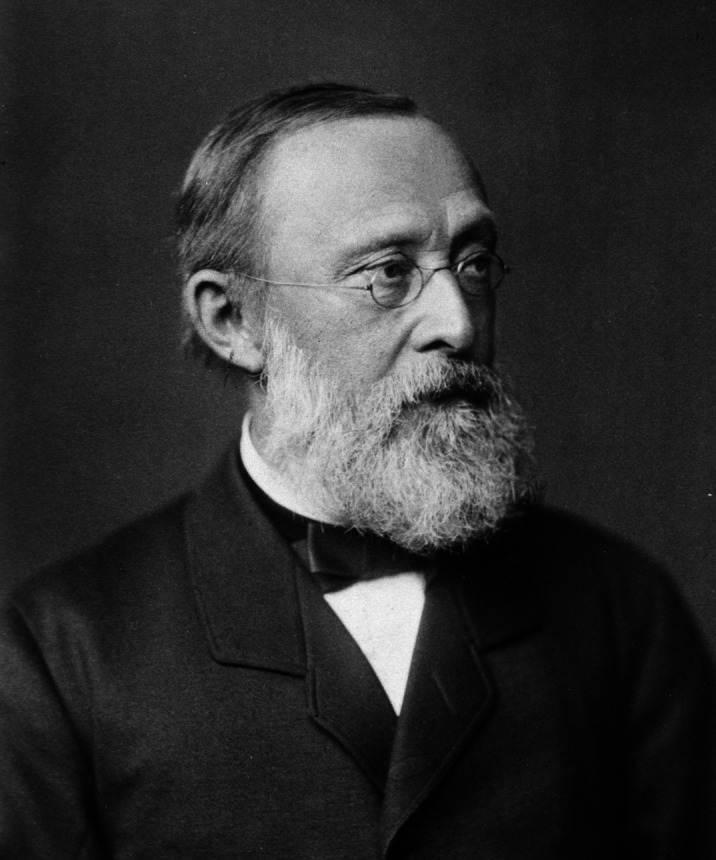
Photo: Rudolf Ludwig Karl Virchow (1821-1902), a German physician known for his advancement of public health. Photo via Wikimedia Commons from the US National Library of Medicine, who believes this item to be in the public domain.

Historically, the close association between health and society was extensively discussed, particularly around the mid–19th century by medical visionaries such as Rudolf Virchow. During his investigation into the typhus epidemic ravaging Upper Silesia, Germany in the late 1840s, Virchow explored the relationship between social conditions, poverty and disease, leading him to the now famous decree: *“Don't crowd diseases point everywhere to deficiencies of society?”.* His prescriptions which focused on addressing the social conditions and resource inequalities of the impoverished population, rather than medical interventions, met with considerable resistance within local government. Whilst this philosophy was not widely welcomed at the time, across the world prominent physicians such as William Chadwick in the United Kingdom, Louis–René Villermé in France and Charles Hastings in Canada were reaching similar conclusions and transforming the face of medicine. This period ushered in sweeping public health reforms which provided clean water, sanitation systems, improved food standards and better living conditions. Collectively these had a transformative effect on population health, improved social conditions and have left a lasting public health legacy still felt today.

Whilst “social medicine” has a history extending farther back to the Hippocratic Corpus and beyond, the academic discipline was developed during the interwar years. In the United Kingdom, interestingly it was a surgeon–John Ryle–who was one of the movement’s pioneering voices. Working alongside other newly appointed professors of social medicine, such as Thomas McKeown, he helped lay down a framework of primary prevention running counter to the prevailing narrative of doctors simply as curers, and health the absence of disease. Contemporaries such as Michael Marmot have since built upon the intellectual foundation of social medicine principles, focusing attention on health inequalities and demanding a reconciliation of social medicine principles with modern medical care. Recognition of the close relationship between health and social circumstance is fundamental to global health discourse and international health practice, however, the implementation of these principles in high–income settings is less commonplace.

For example, in the United Kingdom, the General Medical Council (GMC) which sets the parameters for medical practice (*Good Medical Practice)* [2] continues to rest little weight on public health principles. ‘Public health’ is mentioned only with regards to the duty to *“Respond to requests from organisations monitoring public health”*; the overriding focus is on care for the individual patient. *Tomorrow’s Doctors* [3], the counterpart guidance on medical education lays considerably more emphasis on the broader responsibilities of the doctor. For example, *Outcome 1 (Doctor as Scholar)* paragraphs nine (*Apply Social Science principles, method and knowledge to medical practice*) and ten (*Apply to medical practice the principles, method and knowledge of population health and the improvement of health and health care*). However, *Outcome 2 (Doctor as Practitioner)* gives no attention to the means or considerations in enacting these principles. This discontinuity reflects the broader lack of guidance for clinical doctors in balancing dual responsibilities to the patient and the population. This deficiency has been particularly challenging for collective action problems such as antimicrobial resistance in which the choices of doctors may have adverse effects for others in the community (ie, spread of resistance), even when done in treating the individual in her best interests.

Over recent decades the role of the doctor has retreated from the forefront of medical ethics into the technical sphere of disease. This transition has contributed to an environment in which the role of clinical doctors in improving population health through their daily practice is culturally undermined. Yet, it is also increasingly recognised that no matter how good clinicians are, once patients reach the hospital, or possibly even primary care facilities, it can be too little too late to cure the individual and a sunk cost for the public purse.

Doctors, politicians and the general public in most settings tends to agree that a good health system requires integration between community and hospital care, and that prevention is preferable to cure. Working together to achieve this objective is imperative; whilst priorities may differ, policy must be guided by evidence rather than be blinded by good intentions. This has clear global relevance–balancing public health and immediate clinical needs is a challenge for any health system but these should be clearly set out. Problems as diverse as the Ebola Virus Disease outbreak in West Africa and the rise in antibiotic resistance highlight the intimate relationship between medicine and social context which impacts on the local, national and global level. During the clinical encounter, today’s doctor should of course be wholly committed to the patient in front of them, however, we must face head on the limitations of this approach to effectively improve population health in an era of globalisation and rapid social change.

Acting on this sentiment requires interventions at all stages of medical training, underpinned by an ethos which understands and values these ideals. Achieving this change requires that skills are taught in creative and inspiring ways, and values are rooted in a value system which puts injustice and inequality at the heart of medical practice. General practitioners have a particularly important and opportune role in driving this change. Primary care offers the opportunity to implement primary prevention and improve health, not simply treat disease. Doctors ultimately need time to understand the social determinants of health in their communities–local, national or global–in order to build their skills in epidemiology and public health, and finally to take action and effect change. Therefore, this needs a system wide change which gives doctors space during their training and clinical careers to build relationships across health and other agencies, with encouragement to do so, and emboldened vision to make primary health care for all a reality.

At this testing financial time for health services across the globe, additional duties clearly cannot be given to doctors without the means to fulfil them. However, a first step is recognising the importance of this issue. In the United Kingdom, one clear starting point is for the GMC to establish public health as a core duty of doctors in *Good Medical Practice*. The role of the GMC is to “*protect patients and improve medical education and practice across the UK”* [4]; an overt focus on individual doctor–patient interactions does not maximise this mandate. Incorporating a population health approach into medical practice would help redress the inadvertent adverse effects of the hyper–specialised health care model which modern health systems have increasingly adopted. It could also help ensure medicine is distinguished by an attitude to care for people, not just the ability to diagnose and treat. Addressing the gap between public health practitioners and clinicians in addressing this challenge will thus necessitate a far broader discussion than possible here but should include: the role of nurse–practitioner in primary care and other non–fully trained medical personnel (eg, physician associates) in delivering medical care, the role of the internet and technological innovations in health care provision, and the implications of privatisation on health and social care provision.

John Ryle latterly left a career in surgery to become the world’s first Professor of Social Medicine, amongst his extensive writings he argued that *“as we direct our students, so in large measure must the outlook and method of each new generation of doctors be determined”* [5]. I believe this point must be clearly stated–principles should be enshrined into the work of a doctor, lest the ambition encapsulated in the idea be lost in implementation. This needs a modern re–conceptualised framework of the roles and responsibilities of the doctor which better reflect the multidimensional origins of disease and the greater potential of the medical profession to improve population health. Taking the issues seriously means realising the importance of effective advocacy, and empowering doctors to take on a greater role in protecting and improving the health of populations in an era of chronic disease. Ultimately, by recognising the limitations of clinical medicine to improve the health of our patients and the local populations they are part of, this could nurture an environment in which doctors can take a more prominent role wherever they work to address broader health determinants. 
